# Impact of Iron Supplementation on Growth Performance, Iron Homeostasis and Redox Balance of Suckling Piglets

**DOI:** 10.3390/ani15070924

**Published:** 2025-03-23

**Authors:** Qingwei Meng, Qing Wu, Qiang Zhou, Jiayong Tang, Yong Zhuo, Zhengfeng Fang, Yan Lin, Shengyu Xu, Bin Feng, Lun Hua, Xuemei Jiang, De Wu, Lianqiang Che

**Affiliations:** 1 Department of Strategy Marketing and Technology, Cargill Animal Nutrition, Chengdu 610000, China; qingwei_meng@cargill.com; 2 Key Laboratory for Animal Disease-Resistant Nutrition of China Ministry of Education, Institute of Animal Nutrition, Sichuan Agricultural University, Chengdu 611130, China; 13629038265@163.com (Q.W.); zhouqiang@sicau.edu.cn (Q.Z.); tangjiayong@sicau.edu.cn (J.T.); zhuoyong@sicau.edu.cn (Y.Z.); zfang@sicau.edu.cn (Z.F.); lnyan@sicau.edu.cn (Y.L.); shengyuxu@sicau.edu.cn (S.X.); fengbin@sicau.edu.cn (B.F.); hualun@sicau.edu.cn (L.H.); 71310@sicau.edu.cn (X.J.); wude@sicau.edu.cn (D.W.)

**Keywords:** iron supplementation, growth performance, blood parameters, suckling piglets

## Abstract

This study evaluates the effects of intramuscular injection or oral supplementation of iron on the growth performance, iron homeostasis, and redox status of suckling piglets. A total of 94 newborn piglets, selected from 8 healthy sows, were randomly allocated into 4 treatment groups during the lactation period, receiving injections of normal saline without iron (CON, n = 23), intramuscular dextran injection of bound iron at 2 mL (200 mg Fe) on postnatal day 3 (PND 3) (FeDex, n = 24), oral supplementation from PND 2 to 13 with 10 g of FerkoFer^®^ containing 2.2 g Fe per day (FeOra, n = 23), or both FeDex and FeOra (FeDPO, n = 24). Piglets were weighed individually at birth and PND 21. Blood samples were collected on PNDs 1, 7, 14, and 21. Piglets in the CON group had lower body weight on PND 21 compared with piglets in the FeDex, FeOra, and FeDPO groups (0.46–1.05 kg decrease, *p* < 0.05), while piglets in the FeDPO group had the highest weaning weight across all groups (*p* < 0.05). The levels of hemoglobin (HGB) and serum iron (SI) in FeDPO piglets were significantly higher than in all other groups on PNDs 14 and 21 (*p* < 0.05). Moreover, the red blood cell (RBC) levels in the FeDPO and FeOra piglets were significantly higher than that in FeDex piglets on PND 21 (*p* < 0.05). In addition, serum superoxide dismutase (SOD) and catalase (CAT) activities in both FeOra and FeDPO piglets were significantly higher than those of piglets in CON and FeDex piglets on PND 21 (*p* < 0.05), whereas malondialdehyde (MDA) content in FeDex piglets was higher than that in CON piglets (*p* < 0.05). Collectively, the current results indicate that the combination of intramuscular injection and oral supplementation is a more effective strategy to improve the growth performance of suckling piglets, associated with higher hemoglobin and serum iron levels, as well as the optimal redox status.

## Simple Summary

Iron deficiency is a common problem in suckling piglets since they are born with low iron reserves and sow milk has low iron content. Iron injection alleviates iron deficiency in suckling piglets but inhibits macrophage activity, damages intestinal mucosa, and induces oxidative damage and inflammation. This study evaluated the effects of iron supplementation strategies on the growth performance, iron homeostasis, and redox balance of suckling piglets. The results showed that the combination of oral supplementation and injection of iron was more effective than iron supplementation alone (oral or injection) at improving the growth rate by upregulating the levels of blood hemoglobin and serum iron, associating higher hemoglobin and serum iron levels in piglets with oral iron supplementation.

## 1. Introduction

Iron plays an important role in the transport of oxygen, hematopoiesis, redox balance, and immune function [1,2,3]. Suckling piglets need to retain 7–16 mg of iron daily to support growth [4]. However, sow milk contains an average of only 1 mg of iron per liter [4], which is not sufficient for piglet growth and development when piglets receive milk as the only source of iron [3]. Studies have shown that iron deficiency decreases piglet growth performance and weakens immunity, making piglets more susceptible to pneumonia, gastrointestinal infections, and oxygen transport disorders [5]. However, overloading iron could promote *E. coli* infection, leading to host cell death, diarrhea, and oxidative stress, which disrupts the host immune response and increases the risk of pathogen infection [6,7,8]. Therefore, it is important to supply appropriate amounts of iron to piglets for their growth performance.

According to the previous reports, mother-source iron is not an effective way to improve piglet iron status and growth performance. Intramuscular injection of iron dextran on days 1–3 after birth is widely considered the most effective way to maintain health and prevent anemia in piglets [9]. However, intramuscular injection of a large amount of iron dextran into suckling piglets may perturb the control of the systemic iron metabolic process, heighten iron overload in tissues, decrease fractional iron absorption, and cause acute poisoning with poor efficiency of the antioxidant system and undesirable side effects [10,11,12,13]. Oral iron supplementation has been reported to improve the weaning weight, livability, and serum superoxide dismutase and glutathione peroxidase activities of newborn piglets [14,15]. A single oral dose of 200 mg iron dextran in anemia prevention in piglets was comparable to 200 mg iron as iron dextran given parenterally [16]. Moreover, a double oral administration of 115 mg iron in the form of iron dextran was reported to prevent anemia in piglets, and piglets treated with oral iron dextran achieved higher weight gains than piglets given iron by other methods [17,18].

However, limited research was reported to evaluate the effect of the combination of oral iron administration and intramuscular injection in suckling piglets. Therefore, the objective of this study was to evaluate the effect of different methods of iron supplementation on growth performance, redox balance, and immune status in suckling pigs.

## 2. Materials and Methods

The protocol for all animal procedures was approved by the Animal Care and Use Committee (ACUC) of the Sichuan Agricultural University Institutional Animal Care and Use Committee (SICAU-2022-10).

### 2.1. Animals, Diets, and Experimental Design

A total of 94 newborn piglets (Large White × Landrace × Duroc), selected from 8 healthy sows, were randomly allocated into 4 groups after born on PND 1, receiving an injection of normal saline without iron (CON, n = 23), intramuscular iron dextran injection of bound iron at 2 mL (200 mg Fe) on postnatal day 3 (FeDex, n = 24), oral supplementation with 10 g of FerkoFer^®^ (2.2 g Fe) per day in an individual feeder from postnatal day 2 to 13 (FeOra, n = 23), or both FeDex and FeOra (FeDPO, n = 24). The oral iron-supplemented powder was a commercial product named FerkoFer, which was provided by Biofiber Inc., Gesten, Denmark. The product is a mixture of glycine-iron and ferrous sulfate, containing 220 g/kg Fe. The piglets were given free access to their mothers’ milk in farrowing crates. All piglets had free access to creep feed during the experimental period. Sows had free access to a lactation diet. The farrowing room environment was controlled at approximately 60–80% humidity and a temperature of 28–30 °C. Each piglet was weighed individually on PNDs 1, 7, 14, and 21 at 08:00. The diarrhea of each piglet was observed at 08:30, 12:30, and 16:30 every day and scored according to the diarrhea degree according to the scoring standard (0 = formed feces, 1 = soft feces, 2 = semi-solid feces, 3 = liquid feces). The diarrhea incidence of weaned piglets was determined using the following formula: Diarrhea index = The summation of diarrhea score per piglet per week/7 days/3 times.

### 2.2. Blood Sample Collection

Blood samples were collected from the cervical vein into 5 mL EDTA vacuum tubes for immediate blood biochemical parameter analysis on PNDs 1, 7, 14, and 21 after birth with 10 piglets per treatment. Blood samples were left at room temperature for 30 min, centrifuged at 3000 r/min for 15 min, and stored at –20 °C. On PND 21, the piglets were anesthetized with sodium pentobarbital solution (4%, 40 mg/kg) and euthanized after blood sample collection using carbon dioxide. White blood cell count (WBC), red blood cell count (RBC), hematocrit (HCT), corpuscular volume (MCV), corpuscular hemoglobin (MCH), corpuscular hemoglobin concentration (MCHC), red cell distribution width (RDW), platelet (PLT), platelet count (PLT), platelet volume (MPV), platelet distribution width (PDW), and platelet hematocrit (PCT) were determined with an automatic hematological analyzer (Alfa Basic 16p, Boule Medical AB, Spånga, Sweden).

### 2.3. Serum Iron Concentration

The concentrations of hemoglobin (HGB) in the serum were determined using an automated hematology analyzer (Sysmex K-1000D, Sysmex Inc., Kobe, Japan) on PNDs 1, 7, 14, and 21 after birth. The concentrations of serum iron (SI) in the serum were measured using commercial kits (Serum iron assay kit, No: A039-1, Jiancheng, China) on PNDs 7, 14, and 21 after birth.

### 2.4. Serum Antioxidant Capacity Detection

The serum activities of total superoxide dismutase (SOD), total glutathione peroxidase (GHS-Px), catalase (CAT), and malondialdehyde (MDA) on PNDs 14 and 21 were analyzed using assay kits (Nanjing Jiancheng Bioengineering Institute, Nanjing, China), according to the manufacturer’s instructions.

### 2.5. Statistical Analysis

All data were tested for normal distribution before statistical analysis. Statistical analyses were performed by one-way ANOVA using SPSS 22.0 software (SPSS Inc., Chicago, IL, USA), with each pig as an experimental unit, using the Duncan method to make multiple comparisons. All results are expressed as mean ± standard error, and statistical significance and tendency were considered at *p* < 0.05 and 0.05 ≤ *p* < 0.10, respectively. Graphs were generated using Adobe Illustrator 2021 (Adobe Incorporated, SanJose, CA, USA).

## 3. Results

### 3.1. Growth Performance and Diarrhea Incidence

Piglets in the CON group had lower weaning weights on PND 21 compared with piglets in the FeDPO groups (Table 1, *p* < 0.05), while piglets in FeDPO had the highest weaning weights among all groups (*p* < 0.05). No significant differences in weaning weight were observed between FeDex and FeOra groups. The diarrhea index of piglets did not show statistically significant differences.

### 3.2. Blood Biochemical Parameters

**Figure 1:**
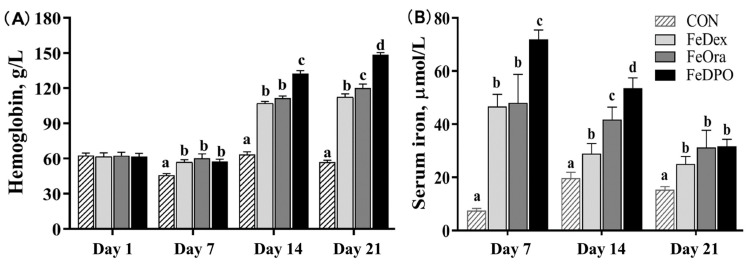
Effects of iron supplementation strategies on hemoglobin and serum iron levels of piglets at different ages. (**A**) Hemoglobin (HGB) (**B**) Serum iron (SI). Values are means and standard errors represented by vertical bars, n = 10 per treatment. ^a–d^ Different letters mean significantly different for different groups at *p* < 0.05. Control treatment (CON); Intramuscular injection of dextran iron treatment (FeDex); Oral iron treatment (FeOra); both FeDex and FeOra iron supplementation treatment (FeDPO).

Piglets in the CON group had lower levels of HGB and SI than those in the FeDex, FeOra, and FeDPO groups on PNDs 7, 14, and 21 (Figure 1, *p* < 0.05). Piglets in the FeDPO group had higher HGB levels on PNDs 14 and 21 than those in the FeDex and FeOra groups (*p* < 0.05), while piglets in the FeOra group had higher HGB levels on PND 21 than those in the FeDex group (*p* < 0.05). FeDPO-treated piglets had higher SI on PNDs 7 and 14 than those in the FeDex and FeOra groups (*p* < 0.05), while piglets in the FeOra group had higher SI on PND 14 than piglets in the FeDex group (*p* < 0.05).

The blood routine parameters are shown in Table 2. Piglets in the CON group had lower levels of RBC, HCT, MCV, MCH, MCHC, MPV, and PDW on PNDs 14 and 21 than those in the FeDex, FeOra, and FeDPO groups (*p* < 0.05). However, CON piglets had higher levels of RDW and PLT on PNDs 14 and 21 than that of the FeDex, FeOra, and FeDPO groups (*p* < 0.05). Piglets in the FeDPO group had higher levels of HCT, MCV, and MCH levels compared to the FeDex and FeOra groups on PNDs 14 and 21 (*p* < 0.05). The RBC levels in FeOra and FeDPO piglets were significantly higher than those in FeDex piglets on PND 21 (*p* < 0.05).

### 3.3. Serum Antioxidant Capacity Parameters

**Figure 2:**
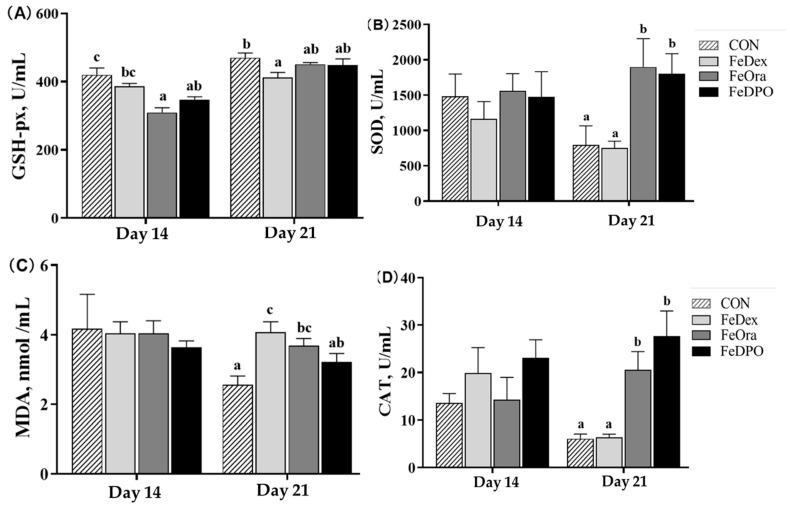
Effect of iron supplementation strategies on redox index of piglets at different ages. (**A**) Total glutathione peroxidase (GSH-Px), (**B**) Total superoxide dismutase (SOD), (**C**) Malondialdehyde (MDA) and (**D**) Catalase (CAT). Values are means and standard errors represented by vertical bars, n = 10 per treatment. ^a–c^ Different letters mean significantly different for different groups at *p* < 0.05. Control treatment (CON); Intramuscular injection of dextran iron treatment (FeDex); Oral iron treatment (FeOra); both FeDex and FeOra iron supplementation treatment (FeDPO).

GSH-px activity in FeOra and FeDPO piglets was lower than in CON piglets on PND 14 (Figure 2, *p* < 0.05). GSH-px activity in FeDex piglets was lower than in CON piglets on PND 21 (*p* < 0.05). SOD and CAT activities in FeOra and FeDPO piglets were significantly increased compared with CON and FeDex piglets on PND 21 (*p* < 0.05). In addition, MDA content in FeDex and FeOra piglets was higher than in CON piglets on PND 21 (*p* < 0.05).

## 4. Discussion

Due to limited fetal iron reserves and low milk iron content, the iron deficiency of newborn piglets is a well-known problem in swine production [3]. Iron-deficient piglets tend to show poor growth, listlessness, rough hair coats, wrinkled skin, and paleness of the mucous membranes [4]. Newborn piglets are often given intramuscular iron dextran (200 mg Fe) at days 2–3 of life to meet their iron needs for better growth and development [14]. Without additional iron supplementation, the piglets will develop anemia 7–10 days after birth [11]. In the current study, piglets in the CON group had lower body weight on PND 21. Through all periods, in this study, HGB and SI levels were lower in the CON group than in piglets with iron supplementation. Several studies have reported that suckling piglets injected with iron supplements have higher weaning weight and daily weight gain [19,20]. Blood HGB levels are a critical indicator of iron status in piglets. Higher HGB levels support oxygen transport and contribute to heavier weaning weights [20,21,22]. HGB levels of 100 g/L of whole blood are considered adequate for piglets, lower than 80 g/L suggest borderline anemia, and 70 g/L or less represent anemia [4,23]. In this study, the HGB levels in the CON group were 47–63 g/L, indicating severe iron deficiency-induced anemia occurred during the whole experimental period. A previous study showed that HGB levels were positively correlated with weaning weight [24].

In this study, however, the piglets in the FeDex and FeOra groups showed similar weaning weights, indicating the growth performance was not markedly affected by the iron supplementation route. Likewise, previous studies showed that no marked differences were observed in weaning weight between oral iron intake with coated ferrous sulfate and intramuscular iron injection [21,25]. Supportively, there was no significant difference in HGB levels on PND 14 between the FeDex and FeOra groups. Moreover, the HGB levels of both FeDex at 101 g/L and FeOra at 111 g/L were higher than normal hemoglobin levels of 100 g/L [18]. However, the SI levels in the FeOra group were significantly higher than the FeDex group on day 14, which may indicate that orally supplementing iron is more effective at maintaining iron nutrition status than injection [26,27,28]. Notably, the heavier weaning weights of piglets in the FeDPO group are in accordance with the higher levels of HGB and HCT compared to the other three groups. It has been reported that the combination of iron supplementation orally and by injection could increase concentrations of serum hemoglobin and hepatic iron and enhance serum iron levels, which are the main biological factors supporting better growth performance [14,29].

In our study, HGB levels in iron-supplemented piglets all increased to >110 g/L over the whole experimental period, which is adequate to prevent anemia with HGB less than 100 g/L [16]. The levels of HCT, MCV, and MCH are sensitive indicators of iron deficiency, and these parameters were all significantly increased on PNDs 14 and 21 in iron-supplemented piglets. A previous study demonstrated that intramuscular iron injection on day 2 after piglet birth can increase the piglet’s HGB, RBC, and SI levels [30]. Even though both were supplemented with iron, the FeOra group showed significantly higher HGB levels than the FeDex group on PND 21, suggesting that oral iron supplementation is a more effective strategy to prevent anemia in suckling piglets [14,31]. RDW level is another sensitive indicator of microcytic hypochromic anemia [32]. The higher RDW levels in CON piglets are also evidence of anemia; in contrast, the lowest RDW levels in FeDPO piglets further indicate that the combination of oral and injected iron is more effective at preventing anemia. In addition, higher PCT indicates clinical symptoms of myelodysplastic syndrome and megaloblastic anemia, while lower PCT indicates regenerative aplastic anemia in piglets [27]. In this study, therefore, the higher PLT and lower MPV levels of the CON group suggest abnormal bone marrow hematopoietic function of piglets [33].

The levels of GSH-px, CAT, and SOD play vital roles in protecting against oxidative damage in piglets [34]. As one of the major secondary decomposition products of lipid oxidation, MDA is closely associated with cell damage and is considered a biomarker for assessing the level of lipid peroxidation [35]. In our study, MDA levels in FeDex piglets were significantly higher on PND 21 than those in CON piglets, while SOD and CAT levels in FeOra and FeDPO piglets were significantly increased. In fact, the injection of iron dextran in suckling piglets did cause acute poisoning with poor efficiency of the antioxidant system [12]. In contrast, the SOD and GSH-px levels in orally iron-supplemented piglets were higher than in the intramuscular iron injection group [14,15]. These findings indicate that oral iron supplementation is beneficial for improving redox status and reduces the risk of oxidative stress by injecting iron dextran in suckling piglets.

## 5. Conclusions

In conclusion, the present results showed that the combination of oral supplementation and injection of iron is more effective at improving growth performance than either oral iron supplementation or intramuscular iron dextran injection, which could be associated with the upregulation of blood hemoglobin and serum iron levels and higher antioxidant capacity.

## Figures and Tables

**Table 1:** Effects of iron supplementation strategies on growth performance and diarrhea index of piglets.

	CON	FeDex	FeOra	FeDPO	*p*
Bodyweight, kg					
Day 1	1.53 ± 0.07	1.50 ± 0.07	1.58 ± 0.06	1.49 ± 0.06	0.75
Day 7	2.82 ± 0.13	2.83 ± 0.14	2.60 ± 0.11	2.63 ± 0.11	0.37
Day 14	4.00 ± 0.19	4.17 ± 0.26	4.29 ± 0.26	4.59 ± 0.22	0.33
Day 21	5.25 ± 0.22 ^a^	5.71 ± 0.31 ^ab^	5.79 ± 0.33 ^ab^	6.30 ± 0.29 ^b^	0.04
Diarrhea index					
Day 1–7	0.02 ± 0.02	0.00 ± 0.00	0.03 ± 0.03	0.00 ± 0.00	0.59
Day 8–14	0.22 ± 0.10	0.11 ± 0.01	0.17 ± 0.13	0.07 ± 0.04	0.67
Day 15–21	0.18 ± 0.05	0.15 ± 0.02	0.15 ± 0.05	0.18 ± 0.07	0.89
Day 1–21	0.14 ± 0.07	0.09 ± 0 01	0.11 ± 0.06	0.08 ± 0.03	0.82

Values are means ± standard error. ^a,b^ Different letters mean significantly different for different groups at *p* < 0.05. Control treatment (CON); Intramuscular injection of dextran iron treatment (FeDex); Oral iron treatment (FeOra); both FeDex and FeOra iron supplementation treatment (FeDPO).

**Table 2:** Effect of iron supplementation strategies on blood routine parameters of piglets at different ages.

	CON	FeDex	FeOra	FeDPO	*p*
PND 14					
WBC, 10^9^/L	9.62 ± 0.99	11.55 ± 1.16	9.90 ± 0.88	10.52 ± 0.65	0.48
RBC, 10^12^/L	4.26 ± 0.14 ^a^	5.24 ± 0.18 ^b^	5.68 ± 0.25 ^b^	5.60 ± 0.10 ^b^	<0.01
HCT, %	21.90 ± 0.82 ^a^	36.42 ± 1.38 ^b^	37.87 ± 1.04 ^b^	42.68 ± 0.56 ^c^	<0.01
MCV, fL	51.53 ± 0.73 ^a^	69.59 ± 1.35 ^b^	67.58 ± 2.30 ^b^	76.38 ± 1.14 ^c^	<0.01
MCH, pg	15.29 ± 0.24 ^a^	22.16 ± 0.4 ^b^	20.03 ± 0.5 ^b^	24.46 ± 0.36 ^c^	<0.01
MCHC, g/L	298.1 ± 4.05 ^a^	319.2 ± 0.96 ^b^	312.64 ± 3.76 ^b^	320.73 ± 1.42 ^b^	<0.01
RDW, %	38.11 ± 0.34 ^b^	18.31 ± 0.42 ^a^	19.19 ± 0.57 ^a^	19.16 ± 0.41 ^a^	<0.01
PLT, 10^9^/L	781.63 ± 26.79 ^b^	614 ± 25.56 ^a^	645.25 ± 50.05 ^a^	669.56 ± 31.71 ^a^	0.01
MPV, fL	7.98 ± 0.21 ^a^	9.49 ± 0.17 ^b^	8.86 ± 0.18 ^b^	8.97 ± 0.08 ^b^	<0.01
PDW	15.65 ± 0.09 ^a^	17.35 ± 0.11 ^b^	17.5 ± 0.19 ^b^	17.69 ± 0.07 ^b^	<0.01
PCT, %	0.58 ± 0.02 ^b^	0.59 ± 0.02 ^b^	0.49 ± 0.02 ^a^	0.58 ± 0.02 ^b^	0.01
PND 21					
WBC, 10^9^/L	9.76 ± 0.82 ^a^	12.79 ± 1.96 ^ab^	18.51 ± 1.67 ^b^	16.6 ± 1.57 ^b^	<0.01
RBC, 10^12^/L	4.03 ± 0.19 ^a^	5.57 ± 0.10 ^b^	6.13 ± 0.13 ^c^	6.20 ± 0.13 ^c^	<0.01
HCT, %	18.09 ± 0.64 ^a^	34.28 ± 1.04 ^b^	36.49 ± 1.13 ^b^	43.56 ± 0.56 ^c^	<0.01
MCV, fL	45.45 ± 1.27 ^a^	61.51 ± 1.46 ^b^	59.73 ± 1.78 ^b^	70.54 ± 1.33 ^c^	<0.01
MCH, pg	14.42 ± 0.77 ^a^	19.39 ± 0.37 ^b^	19.18 ± 0.64 ^b^	23.08 ± 0.36 ^c^	<0.01
MCHC, g/L	299.14 ± 3.01 ^a^	322.8 ± 2.29 ^b^	321.58 ± 3.19 ^b^	328.00 ± 2.75 ^b^	<0.01
RDW, %	36.47 ± 0.60 ^c^	20.23 ± 0.85 ^b^	19.67 ± 0.90 ^b^	17.24 ± 0.42 ^a^	<0.01
PLT, 10^9^/L	1232.4 ± 295.44 ^c^	715.88 ± 33.26 ^a^	857.83 ± 54.42 ^ab^	803.4 ± 78.98 ^ab^	<0.01
MPV, fL	6.87 ± 0.10 ^a^	8.55 ± 0.26 ^b^	8.20 ± 0.15 ^b^	8.75 ± 0.12 ^b^	<0.01
PDW	16.06 ± 0.33 ^a^	17.19 ± 0.19 ^b^	16.95 ± 0.17 ^b^	17.72 ± 0.10 ^b^	<0.01
PCT, %	0.46 ± 0.04 ^a^	0.60 ± 0.03 ^b^	0.60 ± 0.03 ^b^	0.51 ± 0.08 ^ab^	0.04

Values are means ± standard error, n = 10 per treatment. ^a–c^ Different letters mean significantly different for different groups at *p* < 0.05. Control treatment (CON); Intramuscular injection of dextran iron treatment (FeDex); Oral iron treatment (FeOra); both FeDex and FeOra iron supplementation treatment (FeDPO). White blood cell (WBC); Red blood cell count (RBC); Hematocrit (HCT); Mean corpuscular volume (MCV); Mean corpuscular hemoglobin (MCH); Mean corpuscular hemoglobin concentration (MCHC); Red cell distribution width (RDW); Platelet (PLT); platelet count (PLT); Mean platelet volume (MPV); Platelet distribution width (PDW) and Platelet hematocrit (PCT).

## Data Availability

The data presented in this study are available in the article.
